# Effects of Amelogenin on Proliferation, Differentiation, and Mineralization of Rat Bone Marrow Mesenchymal Stem Cells *In Vitro*

**DOI:** 10.1100/2012/879731

**Published:** 2012-04-01

**Authors:** Masanobu Izumikawa, Keijiro Hayashi, Mohammad Ali Akbor Polan, Jia Tang, Takashi Saito

**Affiliations:** Division of Clinical Cariology and Endodontology, Department of Oral Rehabilitation, School of Dentistry, Health Sciences University of Hokkaido, 1757 Tobetsu, Hokkaido 061-0293, Japan

## Abstract

The aim of this study was to clarify the function of amelogenin, the major protein of enamel matrix derivative, on the proliferation, differentiation, and mineralization of cultured rat bone marrow stem cells (BMSCs), toward the establishment of future bone regenerative therapies. No differences in the morphology of BMSCs or in cell numbers were found between amelogenin addition and additive-free groups. The promotion of ALPase activity and the formation of mineralized nodules were detected at an early stage in amelogenin addition group. In quantitative real-time RT-PCR, mRNA expression of osteopontin, osteonectin, and type I collagen was promoted for 0.5 hours and 24 hours by addition of amelogenin. The mRNA expression of osteocalcin and DMP-1 was also stimulated for 24 hours and 0.5 hours, respectively, in amelogenin addition group. These findings clearly indicate that amelogenin promoted the differentiation and mineralization of rat BMSCs but did not affect cell proliferation or cell morphology.

## 1. Introduction

In recent years, with advances in regenerative medicine, the possibility of regenerating mandibular bone, alveolar bone, and dentin has attracted attention. For the induction of alveolar bone formation, enamel matrix derivative (EMD) has been applied in the field of periodontology [1–3]. Hammarström reported that, when mesenchymal cells of the dental follicle were exposed to EMD, a noncellular hard tissue matrix formed at the enamel surface and that the application of porcine EMD in experimental cavities in the roots of incisors of monkeys induced the formation of acellular cementum that was well attached to the dentin [4]. It was reported that porcine EMD was used in patients with severe periodontitis to induce cementogenesis as EMD induces a process that seems to mimic normal odontogenesis [1–3]. Recently, some studies showed that EMD enhances the expression of tissue-specific maturation markers, such as ALPase activity, collagen, and osteocalcin within osseous tissues [5–7]. Moreover, recent studies applied EMD to vital pulp therapy in endodontics [8–10]. A study comparing the effect of EMD with calcium hydroxide as a direct pulp capping agent in pigs [11] demonstrated a significantly more pronounced formation of secondary dentine in teeth treated with EMD.

The major constituent of EMD is amelogenin, a family member of hydrophobic proteins derived from a single gene by alternative splicing and controlled postsecretory processing [12]. It is the major organic component in the enamel matrix of developing teeth and plays an important role in enamel biomineralization [13, 14]. It is specifically detected in ameloblasts, but several studies have also detected it in odontoblasts and might induce the differentiation and maturation of odontoblasts [15–17]. The amelogenin is also known to self-assemble into supramolecular aggregates that form an insoluble extracellular matrix [18] with high affinity for hydroxyapatite and collagens [19]. Many recent studies reported that amelogenin interacts directly with cell types other than cementoblasts [20–22], suggesting that it plays a more direct role in the growth of mesenchymal tissues.

Therefore, we gave importance to the functions of amelogenin, which is a major component of EMD, for the potential of regeneration of hard tissues such as bone and dentin clinically. In the present study, we investigated the effects of amelogenin on proliferation, differentiation, and mineralization in rat bone marrow mesenchymal stem cells (BMSCs) *in vitro*.

We added amelogenin to cultured rat BMSCs and examined the changes of cell morphology and cell number, ALPase activity, and the formation of mineralized nodules. Then, mRNA expression of bone-related proteins osteopontin, osteonectin, osteocalcin, type I collagen, and dentin matrix protein-1 (DMP-1) in BMSCs was detected by using the quantitative real-time RT-PCR method.

## 2. Materials and Methods

### 2.1. Amelogenin

We purchased amelogenin (Hokudo, Sapporo, Japan) having a molecular weight of 25 kDa and purified mainly from enamel of bovine immature tooth. It was adjusted to concentration of 1 mg/mL Dulbecco's phosphate-buffered saline (PBS) after the purchase, and was stored at −80°C until use.

### 2.2. Bone Marrow Mesenchymal Stem Cell (BMSC) Isolation and Culture

All protocols were performed in accordance with the guidelines of the Animal Care Committee of the Health Sciences University of Hokkaido. BMSC isolation and culture were performed according to previously described methods [23]. In brief, we harvested bone marrow from 7-week-old male Fischer 344 rats (Hokudo) by flushing their femoral cavities with Dulbecco's PBS. BMSCs were cultured in *α*-minimal essential medium (*α*-MEM; Invitrogen, Carlsbad, Calif, USA), 15% fetal bovine serum (FBS, MultiSer Trace Scientific, Melbourne, Australia), 100 U/mL penicillin (Invitrogen), and 100 *μ*g/mL streptomycin (Invitrogen). Nonadherent hematopoietic cells were removed, and the medium was replaced. The medium was refreshed every 3 days. The adherent, spindle-shaped BMSC population expanded to >5 × 10^7^ cells within 3–5 passages after the cells were first plated.

### 2.3. Cell Morphology and Proliferation

We compared the morphology and proliferative activity of BMSCs cultured in *α*-MEM and 15% FBS with or without 100 ng/mL amelogenin. In brief, cells (5 × 10^4^ cells/well) were cultured in 24-well plates with culture medium, and changes of cell morphology were observed with phase-contrast microscopy at 2, 5, 8, 11, and 14 days after the addition of amelogenin. Then, cells were harvested and counted with a hemocytometer.

### 2.4. Cell Differentiation

Mineralized nodules were detected by Von Kossa staining [24]. Briefly, cells were fixed with 99.5% ethanol (Wako, Tokyo, Japan) for 10 min, washed with distilled water, and then treated with 5% AgNO_3_ for 15 min, washed again with distilled water, and treated with 5% sodium thiosulfate for 5 min. The specimens were examined under a light microscope.

ALPase activity is considered essential for biomineralization. The cells were washed three times with PBS and sonicated with 1 mL of 0.1-M Tris buffer (pH 7.2) containing 0.1% Triton X-100 (Sigma Chemical, St. Louis, Mo, USA) for 30 sec on ice. Cellular ALPase activity was assayed using p-nitrophenyl phosphate as a substrate. The enzyme activity is expressed as micromoles of p-nitrophenyl produced per minute per milligram of protein. The protein concentration was determined using the Bio-Rad DC protein assay kit (Bio-Rad Laboratories, Hercules, Calif, USA) with albumin as the standard.

### 2.5. Quantitative Real-Time RT-PCR

The cells (1 × 10^5^ cells/dish) were seeded in 60-mm culture dishes. After reaching confluence, amelogenin (0, 10, 100, or 1000 ng/mL) was added to the cells with serum-free medium. After 30 min and 24 h, the cells were collected with TRIzol reagent (Invitrogen). Total cellular RNA was reverse-transcribed using SUPERSCRIPT II (Invitrogen) to synthesize cDNA. PCR was then carried out to measure the mRNA expression of osteopontin, osteonectin, osteocalcin, type I collagen, and dentin matrix protein-1 (DMP-1). The housekeeping gene glyceraldehyde-6-phosphate dehydrogenase (GAPDH) was also measured as a control. Their primer sequences are shown in Table 1.

Quantitative real-time PCR assay was performed with a LightCycler using the double-stranded DNA dye SYBR Green I (Roche Diagnostics, Mannheim, Germany) [25, 26]. Quantification was performed by comparing the levels obtained to standardized samples. The PCR conditions used in the LightCycler are shown in Table 2. The final concentration of MgCl_2_ was 3 mM. A standard curve was constructed by refining PCR products using a High Pure PCR Product Purification Kit (Roche Diagnostics) followed by 10-fold dilutions. Melting curve analysis was also performed after the PCR amplification to confirm the absence of the primer dimer in the PCR products. In addition, the final PCR product on 2% agarose gels migrated as a single band with a sequence identical to that of each protein.

### 2.6. Statistical Analysis

The cell counts and ALPase activities were analyzed by Student's* t-*test and expressed as means ± SD. The gene expressions were analyzed by one-way analysis of variance with Tukey's multiple comparison test. Differences at *P* < 0.05 were considered statistically significant.

## 3. Results

### 3.1. Cell Morphology, Proliferation, Differentiation, and Mineralization

**Figure 1:**
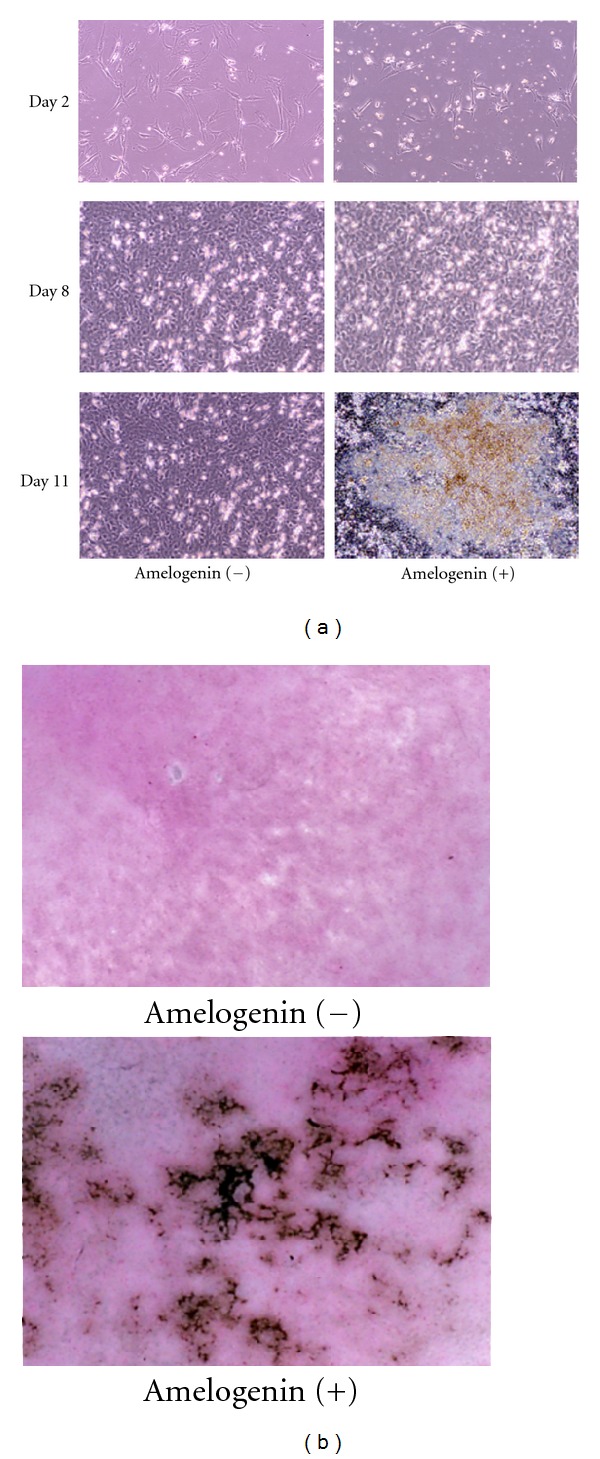
Effects of 100 ng/mL of amelogenin on morphology and mineralization of rat bone marrow mesenchymal stem cells. (a) Phase-contrast features of rat bone marrow mesenchymal stem cells at 2, 8, and 11 days of culture with and without amelogenin (original magnification ×100). (b) Von Kossa staining of rat bone marrow mesenchymal stem cells at 11 days of culture with and without amelogenin (original magnification ×400).

BMSCs with and without exposure to amelogenin were fibroblast-shaped initially and were cubic-shaped after reaching confluence on day 8. In the group with amelogenin, mineralized nodules were detected by Von Kossa staining at day 11 (Figure 1). In the group without amelogenin, mineralized nodules were observed after day 15 (data not shown).

**Figure 2:**
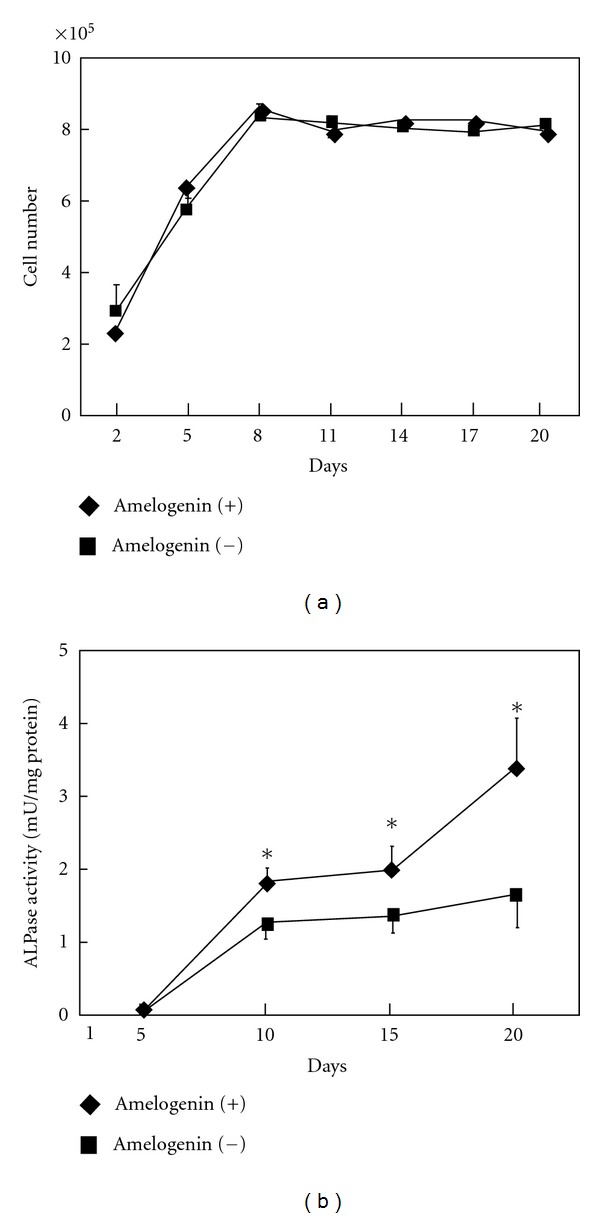
Effects of 100 ng/mL of amelogenin on proliferation and differentiation of at bone marrow mesenchymal stem cells. (a) Time-course changes in numbers of cells cultured with and without amelogenin. (b) Time-course changes in ALPase activity of cells cultured with and without amelogenin. Data represent means ± SD in three samples in three separate experiments. Significantly different from control values (*P < 0.05).

The number of cells did not significantly differ between the groups throughout the experimental period (Figure 2(a)). On the other hand, ALPase activity in the group with amelogenin was significantly higher than that in the group without amelogenin (Figure 2(b)).

### 3.2. Bone-Related mRNA Expression

**Figure 3:**
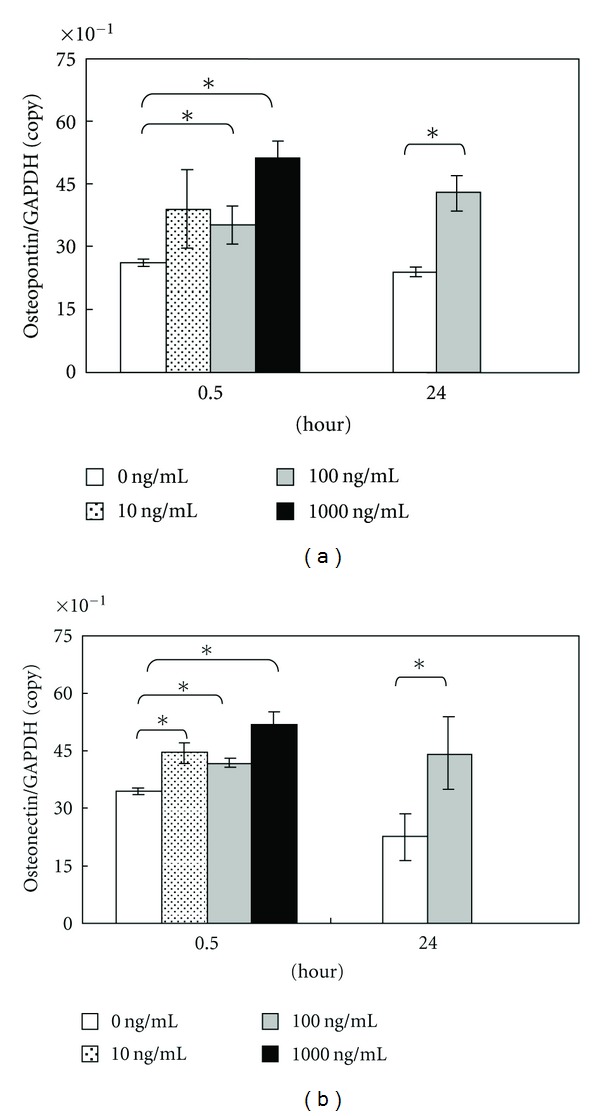
Graph of osteopontin (a) and osteonectin (b) copy number determined using LightCycler. Data represent means ± SD in five samples in three separate experiments. Significantly different from control values (*P < 0.05).

**Figure 4:**
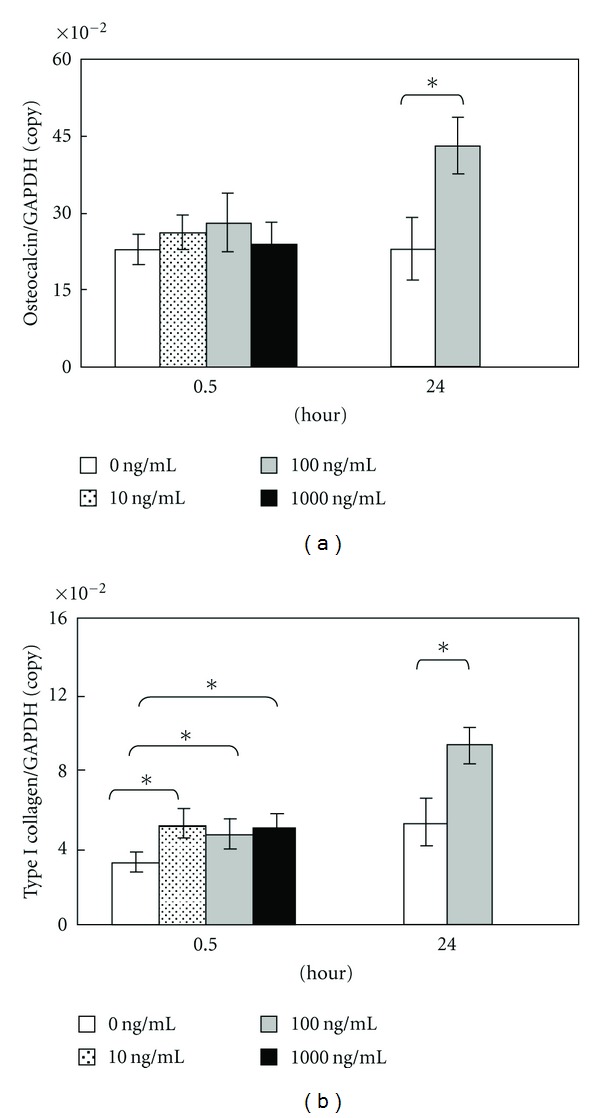
Graph of osteocalcin (a) and type I collagen (b) copy number determined using LightCycler. Data represent means ± SD in five samples in three separate experiments. Significantly different from control values (*P < 0.05).

In quantitative real-time RT-PCR, mRNA expression of osteopontin was promoted significantly by addition of 100 and 1,000 ng/mL of amelogenin for 0.5 hours (Figure 3). As for osteonectin and type I collagen mRNA, the expression was significantly accelerated by the addition of 10, 100, and 1,000 ng/mL of amelogenin for 0.5 hours (Figures 3 and 4). Also, the addition of 100 ng/mL of amelogenin for 24 hours stimulated the expression of osteopontin, osteonectin, and type I collagen mRNA (Figures 3 and 4). Amelogenin addition at a concentration of 100 ng/mL for 24 hours significantly promoted osteocalcin mRNA expression, although the addition of 10, 100, and 1,000 ng/mL of amelogenin for 0.5 hours did not (Figure 4).

**Figure 5:**
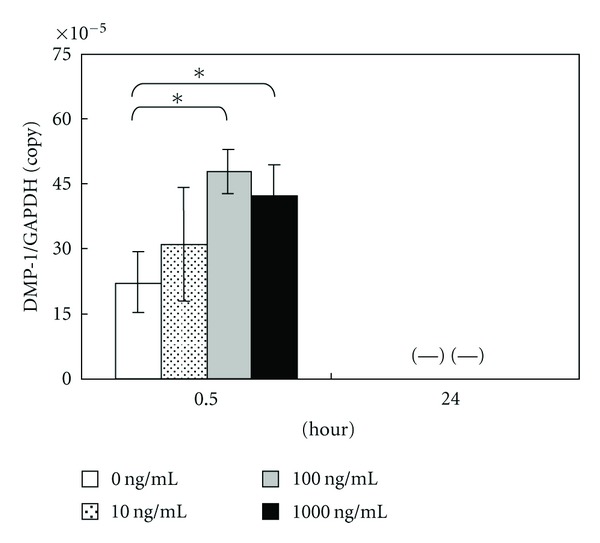
Graph of DMP-1 copy number determined using LightCycler. Data represent means ± SD in five samples in three separate experiments. (—) means not detectable. Significantly different from control values (*P < 0.05).

Moreover, DMP-1 mRNA expression was increased after the addition of amelogenin at a concentration of 100 and 1,000 ng/mL for 0.5 hours, but it was below the detection threshold after 24 hours (Figure 5).

## 4. Discussion

Currently, ectodermal tooth enamel proteins, in the form of a commercial preparation of porcine fetal enamel matrix derivative (EMD), are frequently used in surgical procedures to induce mesenchymal cell differentiation for cementogenesis and periodontal ligament regeneration in patients having severe periodontitis [3]. To date, a wide range of *in vitro* and *in vivo* experimental studies have demonstrated that EMD stimulates the growth of multiple mesenchymal cell types including fibroblasts, cementoblasts, and osteoblasts [4–7]. The study also showed that EMD enhances the expression of tissue-specific maturation markers, such as ALPase activity, type I collagen, osteopontin, and osteocalcin in osteoblastic cell lines [6, 27, 28]. Furthermore, EMD has been reported to induce the formation of mineralized nodules in osteoblastic cell culture [29]. However, it was previously demonstrated that EMD contains TGF-*β*1 or a TGF-*β*-like substance and that EMD rapidly translocates smad2, which is an effector of the TGF-*β* signaling pathway, into the nucleus [30]. Therefore, in the present study, we examined the function of the major (>95%) constituent of EMD, amelogenin, in an *in vitro* experimental system.

We found that 100 ng/mL of amelogenin affected neither the proliferation of BMSCs nor the cell morphology. On the other hand, it stimulated ALPase activity in the cells and the formation of mineralized nodules earlier. The increase in the activity levels of ALP, an intracellular enzyme necessary for mineralization, is considered to be an early marker of cells oriented towards osteogenic production [31]. Matrix-mediated mineral deposition in osteoblasts or osteoblast-like cells was demonstrated by Von Kossa staining. This histological staining is based on the capacity of silver nitrate to specifically react with phosphate in the presence of acidic material, and its positive appearance is considered an expression of the mineralization of bone matrix [32]. Therefore, in the present study, amelogenin functioned as a differentiation-calcification factor to BMSCs rather than a growth factor. A similar effect of apatite/amelogenin coating on titanium on ALPase activity has been reported in human fetal preosteoblasts [33].

We used quantitative PCR to investigate the gene expression of a differentiation marker of osteoblasts in BMSCs in the presence of amelogenin. The commitment of BMSCs to osteogenic differentiation was demonstrated by the expression of osteopontin, osteonectin, osteocalcin, type I collagen, and DMP-1. These proteins are considered as lineage-specific markers of osteoblastic differentiation [34]. Osteopontin is a phosphoprotein member of the SIBLING family that possesses several calcium-binding domains and is associated with cell attachment, proliferation, and mineralization of extracellular matrix into bone, synthesized by bone-forming cells [34, 35]. Osteonectin, also known as SPARC, is a calcium- and collagen-binding extracellular matrix glycoprotein abundantly expressed in bone undergoing active remodeling [36]. Osteocalcin binds with high affinity to hydroxyapatite crystals, the key mineral component of bone, and regulates bone crystal growth [37]; it is the latest secreted extracellular matrix protein to be identified. Type I collagen is the major organic component of bone matrix produced by osteoblasts. It functions as a scaffold of mineralization in bone. In the present study, we found that the expressions of osteopontin mRNA was accelerated by addition of 100 and 1,000 ng/mL of amelogenin for 0.5 hours as well as by the addition of 100 ng/mL of amelogenin for 24 hours, and that as for osteonectin and type I collagen mRNA expression was promoted by addition of every amelogenin concentration tested (10,100 and 1,000 ng/mL) for 0.5 hours as well as by the addition of 100 ng/mL of amelogenin for 24 hours. On the other hand, stimulation of osteocalcin mRNA expression by 100 ng/mL of amelogenin required 24 hours. These results are consistent with the reports described above. Moreover, our previous study demonstrated that amelogenin enhanced mRNA expressions of BMP-2 and BMP-4 in rat BMSCs [38]. Chen et al. reported that BMP-2 enhances BMP-3, BMP-4, type I collagen, osteopontin, and osteocalcin mRNA expression and promotes ALPase activity as a differentiation marker in rat calvarium osteoblasts [39]. Thus, differentiative induction from BMSCs into osteoblasts by the addition of amelogenin is thought to be mediated by BMP expression.

DMP-1, an acidic phosphorylated extracellular matrix protein [40], is expressed in odontoblasts that secrete matrix proteins to form dentin. *In vitro *studies suggest that overexpression of *Dmp-1 *induces the differentiation of mesenchymal cells to odontoblast-like cells and enhances mineralization [41] and that DMP-1 can bind to Ca^2+^ and initiate mineral deposition *in vitro *[42]. Moreover, DMP-1 is shown to be a bone matrix protein expressed in osteoblasts and osteocytes and is assumed to play a role in bone mineral homeostasis due to its high calcium ion-binding capacity [43]. As shown in a recent report that a lack of DMP-1 gave rise to rickets or osteomalacia in mice [44], a possible role of osteocytes mediating DMP-1 appears to be the local regulation of mineralization. Thus, it is not specific in dentin. In the present study, DMP-1 mRNA expression was increased by amelogenin addition at concentrations of 100 and 1,000 ng/mL for 0.5 hours. However, the level of DMP-1 mRNA expression was lower than those of other genes. Especially, it was below the detection threshold after 24 hours. Thus, the induction may have been caused by osteoblastic differentiation rather than odontoblastic differentiation.

Our results clearly showed that amelogenin promoted differentiation and mineralization of cultured rat BMSCs but did not affect cell proliferation or cell morphology. Further studies are necessary to elucidate the mechanism of the induction of osteoblastic differentiation of BMSCs by amelogenin and also confirm the differentiation in animal models. *In vivo* experiments will be required in order to clarify the significance of amelogenin-induced hard-tissue regeneration.

## Figures and Tables

**Table 1:** Primer sequences for target cDNAs.

Target cDNA	Primer sequence
GAPDH (200 bp)	Upstream: 5′-TCC ATG ACA ACT TTG GTA TCG-3′Downstream: 5′-ATG AGT CCT TCC ACG ATA CCA-3′
Osteopontin (220 bp)	Upstream: 5′-CTC AGA GGA GAA GGC GCA TTG-3′Downstream: 5′-TCT CTG CAT GGT CTC CGT CGT -3′
Osteonectin (225 bp)	Upstream: 5′-GTC CTG GTC ACC TTG TAC GAG-3′Downstream: 5′-GGG ACA GGT ACC CAT CAA TCG -3′
Osteocalcin (175 bp)	Upstream: 5′-AAG GTG GTG AAT AGA CTC CG-3′Downstream: 5′-AAA CGG TGG TGC CAT AGA TG-3′
Type I collagen (181 bp)	Upstream: 5′-TGC CGT GAC CTC AAG ATG T-3′Downstream: 5′-TGG GGT TTG GGC TGA TGT A-3′
DMP-1 (163 bp)	Upstream: 5′-AGC ATT CCT CTA ATC CAG TT-3′Downstream: 5′-CTG ATC TAA ACA AGT GCC AT-3′

**Table 2:** Conditions of quantitative PCR using LightCycler.

Target cDNA	PCR conditions			
GAPDH	95°C 10 min	(35 cycles) 95°C 10 sec 62°C 5 sec 72°C 15 sec	67°C 10 sec	40°C 60 sec
Osteopontin	95°C 10 min	(30 cycles) 95°C 10 sec 63°C 5 sec 72°C 15 sec	78°C 10 sec	40°C 30 sec
Osteonectin	95°C 10 min	(30 cycles) 95°C 10 sec 61°C 5 sec 72°C 15 sec	66°C 10 sec	40°C 30 sec
Osteocalcin	95°C 10 min	(35 cycles) 95°C 10 sec 62°C 10 sec 72°C 05 sec	70°C 15 sec	40°C 30 sec
Type I collagen	95°C 10 min	(40 cycles) 95°C 10 sec 62°C 10 sec 72°C 07 sec	70°C 15 sec	40°C 30 sec
DMP-1	95°C 10 min	(40 cycles) 95°C 10 sec 62°C 10 sec 72°C 07 sec	70°C 15 sec	40°C 30 sec
